# Frequency of Maternal and Newborn Birth Outcomes, Lima, Peru, 2013

**DOI:** 10.1371/journal.pone.0116102

**Published:** 2015-03-25

**Authors:** Adriane Wynn, Jeanne Cabeza, Kristina Adachi, Jack Needleman, Patricia J. Garcia, Jeffrey D. Klausner

**Affiliations:** 1 Fielding School of Public Health, Department of Health Policy and Management, University of California Los Angeles, Los Angeles, California, United States of America; 2 Center for AIDS Research and Education (CARE), Los Angeles, California, United States of America; 3 David Geffen School of Medicine, University of California Los Angeles, Los Angeles, California, United States of America; 4 School of Public Health and Administration (FASPA), Lima, Peru, Universidad Peruana Cayetano Heredia (UPCH), Lima, Peru; 5 Division of Infectious Diseases, Center for World Health and Department of Epidemiology, David Geffen School of Medicine and Fielding School of Public Health, University of California Los Angeles, Los Angeles, California, United States of America; Medical University of Vienna, AUSTRIA

## Abstract

**Objective:**

This study describes the pregnancy and birth outcomes at two hospitals in Lima, Peru. The data collection and analysis is intended to inform patients, providers, and policy makers on Peru’s progress toward achieving the Millennium Development Goals and to help set priorities for action and further research.

**Methods:**

Data were collected retrospectively from a sample of 237 women who delivered between December 2012 and September 2013 at the Instituto Nacional Materno Perinatal or the Hospital Nacional Arzobispo Loayza. The outcomes were recorded by a trained mid-wife through telephone interviews with patients and by review of hospital records. Associations between participant demographic characteristics and pregnancy outcomes were tested with Chi-squared, Fisher’s exact, or Student’s t-test.

**Results:**

Over 37% of women experienced at least one maternal or perinatal complication, and the most frequent were hypertension/preeclampsia and macrosomia. The women in our sample had a cesarean section rate of 50.2%.

**Conclusion:**

Maternal and perinatal complications are not uncommon among women in the lower socioeconomic strata of Lima. Also, the high cesarean rate underpins the need for a more comprehensive understanding of the indications for cesarean section deliveries, which could help reduce the number of unnecessary procedures and preventable complications.

## INTRODUCTION

Since the adoption of the Millennium Development Goals (MDG) by the United Nations in 2000, much progress has been made toward improving maternal, infant, and child outcomes. According to a 2012 World Health Organization (WHO) report, maternal deaths have dropped from 543,000 annual deaths in 1990 to 287,000 in 2010; deaths among children under five years of age have declined from 12 million annual deaths in 1990 to 7.6 million in 2010.[1] Nevertheless, more work is necessary to address key morbidity and mortality indicators that remain stagnant and undocumented. Studies have shown that more than 2.6 million stillbirths and 3.8 million neonatal deaths continue to occur annually.[2,3] Neonatal deaths account for approximately 40% of all child deaths.[4] Further, 15 million babies are estimated to be born preterm every year and over one million babies die annually from preterm birth complications.[5]

In addition, poor birth outcomes strain health care systems and cause economic burdens. For example, preterm birth is associated with long-term, adverse health consequences, including higher risks for cerebral palsy, learning disabilities, and respiratory diseases compared to individuals born at term.[6] Although, these outcomes frequently result from complex medical, psychological, social and environmental factors, advancements can be made with current, evidence-based interventions.[5] Many Latin American countries, including Peru, have recognized the importance of improving birth outcomes and are looking for solutions in prevention and care.[7]

In light of global efforts to collect and improve maternal and infant indicators of morbidity and mortality, we aimed to describe the birth outcomes at two large, urban hospitals in Lima, Peru. These hospitals serve a large proportion of women in metropolitan Lima and the results of our analysis may be generalizable to women in the lower socioeconomic strata of the city. The collection of current data is intended to inform patients, providers, and policy makers on Peru’s progress toward achieving the Millennium Development Goals and to help set priorities for action and further research.

## METHODS

This is an analysis of maternal and infant outcome data obtained from two, large hospitals located in Lima, Peru. Data correspond to deliveries that occurred from December 2012 to September 2013. The Instituto Nacional Materno Perinatal (INMP) is Peru’s largest reference maternity hospital and averages more than 13,000 births annually. The Hospital Nacional Arzobispo Loayza (HNAL) is also a national hospital with a large obstetrics service. The hospitals serve an urban, primarily low-income population.

The maternal and infant outcome data were collected from a convenience sample of 237 (39.5%) women who were among the 600 participants in a study of *Chlamydia trachomatis* infection screening and treatment in pregnancy. The initial sample was collected over the course of two months beginning in December 2012. Pregnant women who were at least 16 years of age were approached during their first routine antenatal care clinic visit, and 600 (93.8%) of 640 were enrolled after providing informed consent.[8] Following completion of the *Chlamydia trachomatis* study, the maternal and infant outcomes of 237 pregnancies were collected retrospectively by a trained mid-wife through telephone interviews with patients and by review of hospital records. The data were captured and stored electronically. Written consent was obtained from study participants seeking care at Instituto Nacional Materno Perinatal, and verbal consent was obtained from participants at Hospital Nacional Arzobispo Loayza. In the case of Hospital Nacional Arzobispo Loayza, the institution gave approval for the written document to be signed only by the study midwife. This procedure was used in order to protect the confidentiality of the study participants who provided private information about sexually transmitted diseases. Additionally, research staff used a minor assent form for patients ≥ 16 years and <18 years, which was signed in both institutions by the tutor or guardian. In the case of the Hospital Nacional Arzobispo Loayza, the minor did not sign (verbal consent) and in the case of the Instituto Nacional Materno Perinatal, the minor signed (written consent). The consents used in both hospitals are written documents identified by the participant's study number and are stored in the University of California, Los Angeles’ locked office in Lima, Peru. The study protocol and consent procedures were approved by the institutional review boards of the University of Peruana Cayetano Heredia, the local hospitals, and the University of California, Los Angeles.

The outcome variables collected include gestational age at delivery, method of delivery, as well as 18 other types of maternal complications of pregnancy and/or delivery and 12 perinatal complications. Stillbirths were diagnosed in a fetus delivered with no signs of life on or after 28 weeks of gestation.[9] Preterm labor was defined in those pregnant women with regular uterine contractions and cervical changes prior to 37 weeks gestation. Premature rupture of membranes was defined in those pregnant women with rupture of membranes prior to uterine contractions. Preterm delivery was diagnosed in those pregnant women in whom delivery occurred before 37 weeks, one day of gestational age.[7] Low birth weight was defined in those infants less than 2500g and small for gestational age in those infants with a weight below the 10th percentile for gestational age. Macrosomia was defined in those with a birth weight greater than 4000g.[10]

To test the association between participant demographic characteristics and pregnancy outcomes we used Chi-squared, Fisher’s exact test, or Student’s t-test. Analyses were completed using Stata 12.1 (Stata Corporation, College Station, TX).

## RESULTS

Two hundred and sixty-eight women were contacted following completion of the *Chlamydia trachomatis* screening study; 237 (88.4%) agreed to provide information on birth outcomes. Demographic information about the participants is shown in Table 1. Among the outcome study participants, 110 (46.4%) were from Instituto Nacional Materno Perinatal and 127 (53.6%) from Hospital Nacional Arzobispo Loayza. The median age was 27 years (range 16–47) with a median of two lifetime partners (range 1–50). Most of the women were married or cohabiting (75.1%), had at least a high school education (95.8%), and nearly two-thirds had a previous pregnancy (61.5%). Those characteristics are similar across the two hospital samples (Table 1).

**Table 1:** Characteristics of study participants, total and stratified by hospital site, Lima, Peru, 2013 (N = 237).

	Total sample (N = 237)	Hospital 1 (N = 110)	Hospital 2 (N = 127)
Characteristics	N (%)	N (%)	N (%)
Median age in years (range)	27 (16–44)*	27 (17–44)*	27 (16–43)*
Education			
None/Elementary	10 (4.2)	5 (4.5)	5 (3.9)
High School	143 (60.3)	57 (51.8)	86 (67.7)
University/Tech	84 (35.4)	48 (43.6)	36 (28.4)
Partnership status			
Single/Separated/Widowed	50 (21.1)	16 (14.6)	34 (26.8)
Married/Cohabitating	187 (78.9)	94 (85.5)	93 (73.2)
Parity			
First Pregnancy	84 (35.4)	44 (40)	40 (31.5)
> Second Pregnancy	153 (64.6)	66 (60)	87 (68.5)
Sexual History			
Lifetime no. partners (range)	2 (1–10)*	2 (1–10)*	2 (1–10)*
Prior diagnosis of syphilis	2 (0.8)	2 (1.8)	0 (0)
Prior diagnosis of HIV	0 (0)	0 (0)	0 (0)
Condom use in last encounter	13 (5.4)	2 (1.8)	11 (8.6)

Note: Hospital 1 is the Instituto Nacional Materno Perinatal and Hospital 2 is the Hospital

Nacional Arzobispo Loayza.

* Mean (range)

As seen in Table 2, among the 237 participants, the median gestational age at delivery was 38 weeks and 224 (94.5%) delivered at term. Forty-four (18.6%) women experienced some type of maternal complication during pregnancy and/or delivery. The most common complications were hypertension or preeclampsia (4.6%) and premature rupture of membranes (3.8%). Additionally, seven (3.0%) women experienced two complications and 15 (6.3%) women with a maternal pregnancy or delivery complication also had a perinatal complication event. There were no cases of maternal HIV infection in our sample, but two (0.8%) women had a history of prior syphilis infection. The lack of HIV infection was expected given the low prevalence of HIV (1.2%) found in the larger sample of women who participated in the Chlamydia trachomatis study.[8] Further, this finding is similar to the results of a country-wide survey, which showed that the prevalence of HIV in young women in Peru is 0.1%.[11] Finally, an assessment of delivery methods showed that cesarean sections were performed for 119 (50.2%) women.

**Table 2:** Birth outcomes of participants stratified by hospital, Lima, Peru, 2013 (N = 237).

	Total sample (N = 237)	Hospital 1 (N = 110)	Hospital 2 (N = 127)
Delivery Outcomes	N (%)	N (%)	N (%)
Gestational age (median, range)	38 (11–42)*	38 (33–42)*	38 (11–42)
Deliveries at term (≥ 37 weeks gestation)	224 (94.9)	103 (94.5)	121 (95.3)
Prematurity (<37 weeks gestation)	12 (5.1)	6 (5.5)	6 (4.7)
Medical or Surgical Termination of Pregnancy	2 (0.84)	0 (0)	2 (1.6)
Stillbirth	1 (0.4)	1 (0.9)	0 (0)
**Method of Delivery**			
Cesarean Section	119 (50.2)	55 (50)	64 (50.4)
Vaginal Delivery	117 (49.4)	55 (50)	62 (48.8)
**Complications**			
No Complications	148 (62.5)	58 (52.7)	90 (70.9)
No Maternal Complications (pregnancy/delivery)	193 (81.4)	85 (77.3)	108 (85.0)
No Perinatal Complications	179 (75.5)	76 (69.1)	103 (81.1)
**Maternal Complications**			
Hypertension/ preeclampsia	11 (4.6)	4 (3.6)	7 (5.5)
Premature rupture of membranes	9 (3.8)	5 (2.1)	4 (3.1)
Intrauterine growth retardation	3 (1.3)	3 (2.7)	0 (0)
Placenta previa	3 (1.3)	2 (1.8)	1 (0.8)
Hypothyroidism	3 (1.3)	1 (0.9)	2 (1.6)
Endometritis	2 (0.8)	2 (1.8)	0 (0)
Gastroschisis	2 (0.8)	2 (1.8)	0 (0)
Hyperthyroidsm	2 (0.8)	1 (0.9)	1 (0.8)
Hemolysis Elevated Liver Enzymes Low Platelet Count (HELLP) syndrome	2 (0.8)	1 (0.9)	1 (0.8)
Medical or surgical termination	2 (0.8)	0 (0)	2 (1.6)
Positive for syphilis infection (chart review)	2 (0.8)	2 (1.8)	0 (0)
Oligohydramnios	2 (0.8)	2 (1.8)	0 (0)
Urinary Tract Infection	2 (0.8)	2 (1.8)	0 (0)
Hydatid cyst	1 (0.4)	0 (0)	1 (0.8)
Postpartum hemorrhage	1 (0.4)	1 (0.9)	0 (0)
Uterine rupture	1 (0.4)	1 (0.9)	0 (0)
Venous thrombosis	1 (0.4)	0 (0)	1 (0.8)
Positive for HIV infection (chart review)	0 (0.0)	0 (0)	0 (0)
**Perinatal Complications**			
Macrosomia	23 (9.7)	14 (12.7)	9 (7.1)
Malposition	11 (4.6)	7 (6.36)	4 (3.2)
Nuchal Cord	9 (3.8)	6 (2.5)	3 (2.4)
Hyperbilirubinemia	4 (1.7)	2 (1.8)	2 (1.6)
Low birth weight	4 (1.7)	1 (0.9)	3 (1.7)
Conjuntivitis	3 (1.3)	2 (1.8)	1 (0.8)
Acute respiratory distress	2 (0.8)	2 (1.8)	0 (0)
Congenital malformations	2 (0.8)	0 (0)	2 (1.6)
Fetal bradycardia	2 (0.8)	0 (0)	2 (1.6)
Cord prolapse	1 (0.4)	1 (0.9)	0 (0)
Cyst in CNS	1 (0.4)	1 (0.9)	0 (0)

*Mean (range)

Perinatal complications were recorded in 58 (24.5%) cases, and the most commonly experienced complications were macrosomia (9.7%), malposition (4.6%), birth presentation with a nuchal umbilical cord (3.8%), and low birth weight (1.7%). One infant experienced two complications, which were malposition and macrosomia. As mentioned above, 12 (5.1%) babies were delivered preterm. Among these 12, six experienced perinatal complications, including low birth weight and congenital malformations.

Student’s t-tests revealed that age is associated with a number of maternal and perinatal complications. Women who experienced perinatal complications had a statistically significant (p<0.015) higher mean age (M = 29, SD = 7.4) than women with no perinatal complications (M = 27, SD = 6.7). In terms of specific complications, women who had deliveries complicated by fetal malposition are older on average (M = 32, SD = 8.1) compared to women without this complication (M = 27, SD = 6.8), p = 0.03). Also, there is a statistically significant difference in the age of those who experienced hypertension or preeclampsia (M = 32, SD = 5.6) compared to those that did not (M = 27, SD = 6.9), p = 0.02.

Women who had a cesarean delivery were older on average (M = 29, SD = 7.2) compared to women who had a vaginal delivery (M = 25, SD = 6.3), and this difference is statistically significant (p<0.000). The distribution of cesarean deliveries did not differ significantly across education level or partnership status. Fisher’s exact tests revealed that cesarean deliveries are positively associated with complications such as premature rupture of membranes (p = 0.036), fetal malposition (p = .001), and macrosomia (p = 0.026). Among the 119 women that had a cesarean delivery, eight (6.7%) had birth presentation with a nuchal umbilical, eight (6.7%) experienced a premature rupture of membranes, 11 (9.2%) had fetal malposition, and 17 (14.3%) had macrosomia (Table 3).

**Table 3:** Possible clinical indicators for cesarean stratified by cesarean delivery (N = 237).

	Cesarean Delivery (N = 119)	Vaginal Delivery (N = 117)	
Complications	N (%)	N (%)	Fisher’s Exact p-value
None	49 (41.2)	99 (84.6)	P<0.0001
**Maternal Complications**			
Premature rupture of membranes	8 (6.7)	1 (0.9)	P = 0.036
**Perinatal Complications**			
Macrosomia	17 (14.3)	6 (5.1)	P = 0.026
Malposition	11 (9.2)	0 (0)	P = 0.001
Nuchal Cord	8 (6.7)	1 (0.9)	P = 0.036
Acute respiratory distress	2 (1.7)	0 (0)	P = 0.498
Fetal bradycardia	2 (1.7)	0 (0)	P = 0.498
Cord prolapse	1 (0.8)	0 (0)	P = 1.000

## DISCUSSION

We completed a survey of pregnancy and birth outcomes among women delivering at two large maternity hospitals, located in the urban city of Lima, Peru, an upper middle-income country.[11] The rates of adverse pregnancy outcomes found in our sample were slightly lower than those found in previous studies in South America and Peru. For example, a WHO 2010 systematic review of data collected between 2002 and 2007 found the preterm birth rate in South America to be 7.9%, which is higher than the 5.1% found in our sample.[6] Further, an analysis of data from a WHO global survey on maternal and perinatal health (2004–2008), found that 0.6% of women in Peru experienced neonatal deaths, and 1.6% experienced stillbirth.[12] As described above, the stillbirth rate in our sample was 0.4%. The frequency of macrosomia was higher in our sample (9.7%) than the previously reported rates in Peru in the WHO global survey (6–8%).[10] Risk factors associated with macrosomia include older age, higher body mass index, and diabetes. As in our study, prior reports show that macrosomia significantly increases the risk of cesarean section.[10]

Our finding that 50% of women in those urban public hospitals in Lima delivered by cesarean section was striking. This figure is similar to the rate in metropolitan Lima found by the Demographic and Health Surveys (DHS) project, which conducts nationally representative household surveys on fertility, contraception, maternal and child health and nutrition in Peru.[13] The DHS survey found that country-wide cesarean sections have increased from 21.4% to 25.3% between the 2009 and 2012 reporting periods.[13] Those high rates are not equally distributed among all women in Peru. Cesarean sections were performed in 48.6% of women in the upper quintile of wealth and in 44.1% of women with higher education. The rates also vary by geographic area. For example, northwestern Peru had the highest rate of cesareans at 48.7% followed by metropolitan Lima at 41%. The lowest rate, 8.6%, was found in the Andean region in Huancavelica.[13]

The high number of cesarean sections found in our sample suggests the need for further investigation. As demonstrated in Table 3, our sample provides some evidence that not all of the cesareans were clinically indicated. Among the 119 women who delivered by cesarean, 49 (41.2%) did not experience complications during pregnancy. When cesareans occur without clinical indication, not only are costs significantly higher, but these deliveries are associated with increased risk for adverse short-term complications.[14] As such, some groups like the American Congress of Obstetricians and Gynecologists recently issued guidelines to decrease the overuse of cesareans among first time mothers.[15] Further, the WHO recommends that health facilities with high rates of cesareans should undertake efforts to decrease the number that are not medically necessary.[16] While this study detected a high rate of cesarean deliveries, further research is needed to better understand the determinants and health outcomes associated with the increasing use of this procedure in Peru and other upper middle income countries.

It is important to note that our research findings are subject to a number of limitations. The primary limitations of our study are the use of a non-systematic sample and potential non-response bias. Individuals who reported pregnancy and birth outcome results were not selected at random and may not fully represent the population of women giving birth at the study hospitals.[17] For example, it is possible that our sampling captured women who were more likely to have higher income, fewer complications, and more likely to have a cesarean section. A second limitation is that 88% of women contacted agreed to participate in the study, and we do not fully know how these participants differ from the original sample. In order to evaluate possible non-response bias, we compared the demographic statistics of our sample with those that did not respond. A simple examination of the demographic characteristics of non-respondents showed that they were similar in age, education level, and marital status as the women that participated, suggesting that response bias might not be large. Our results are also strengthened by the fact that the Instituto Nacional Materno Perinatal and the Hospital Nacional Arzobispo Loayza serve a large proportion of women in Lima and that our results are consistent with those found by the most recent DHS survey.

## CONCLUSION

In conclusion, adverse pregnancy and birth outcomes among women in Peru are not unusual. The most common maternal complication was hypertension/preeclampsia and the most common newborn complication was macrosomia followed by fetal malposition, fetal nuchal cord, and premature rupture of membranes. The rates of macrosomia and cesarean section were associated and higher than those found in previous studies of Peru. Finally, a more comprehensive understanding of the indications for cesarean section deliveries might help reduce the number of unnecessary procedures and the associated preventable complications in mothers and infants. Regular monitoring of pregnancy and birth outcomes should be an integral component of obstetric and neonatal care in order to facilitate more rapid reductions in maternal and newborn morbidity and mortality.
